# The Effects of Unconventional Feed Fermentation on Intestinal Oxidative Stress in Animals

**DOI:** 10.3390/antiox13030305

**Published:** 2024-02-29

**Authors:** Xiao Lian, Mingyu Shi, Ying Liang, Qinlu Lin, Lingyu Zhang

**Affiliations:** 1 College of Food Science and Engineering, Central South University of Forestry and Technology, Changsha 410004, China; 20211200637@csuft.edu.cn (X.L.); 20230100090@csuft.edu.cn (M.S.); liangy@csuft.edu.cn (Y.L.); t20081475@csuft.edu.cn (Q.L.); 2 CAS Key Laboratory of Agro-Ecological Processes in Subtropical Region, Institute of Subtropical Agriculture, Chinese Academy of Sciences, Changsha 410125, China

**Keywords:** unconventional feed, fermented probiotics, gut health, oxidative stress, antioxidant

## Abstract

Unconventional feed, which is abundant in China, contains anti-nutritional factors and toxins; however, these can be greatly reduced with microbial fermentation, thus improving the nutrient content of the feed, enhancing animal appetites, and ultimately significantly improving the intestinal health and growth performance of animals. When oxidative stress occurs, fermented feed can effectively reduce the damage caused by stress to the gastrointestinal tract, accelerate the removal of gastrointestinal abnormalities, improve the ability to resist intestinal stress, and ensure the efficient production of animals. This review introduces the application of unconventional fermented feed in animal production, and expounds upon the function of unconventional fermented feed in animals with oxidative stress symptoms, so as to provide a theoretical reference for the development and application of unconventional fermented feed in antioxidative stress reduction.

## 1. Introduction

Unconventional feed refers to feed that is different in terms of the raw material source or preparation process compared to conventional feed. This kind of feed is usually obtained from diversified raw materials such as agricultural and sideline products, aquatic product by-products, and industrial by-products, which are obtained through special processing or treatment; China possesses rich resources of such materials [1,2]. However, the nutrient composition of unconventional feed is complex, and it has shortcomings such as a high content of anti-nutritional factors and poisons, poor palatability, unstable nutrient composition, and significant quality variations [3]. Therefore, the comprehensive utilization level of unconventional feed is low, resulting in a waste of resources, environmental pollution, and other problems. At present, unconventional feed processed through microbial fermentation technology, crushing, heating, hydrolysis, drying, and other methods, in order to degrade the anti-nutritional factors, toxins, crude fiber, lignin, and other substances present in it and reflect its high nutritional value [4] in terms of protein, minerals, and trace elements required for livestock supplementation, is called “special feed” or “alternative feed”. Therefore, unconventional feed is often used, in part, to replace conventional feed to reduce feed costs, improve the economic value, and achieve sustainable development in the feed industry. In recent years, the popularity of unconventional feeds has gradually increased.

With the development of the economy and the improvement of people’s living standards, agriculture and animal husbandry have developed rapidly, and the problem of “human and livestock competing for food” is still severe due to the increase in demand for feed and the insufficient supply [5]. At present, “bans of antibiotics” in feed have become an important trend in the development of the livestock and poultry industry, along with studies on several antibiotic alternatives, such as probiotics, antimicrobial peptides, Chinese herbal additives, plant-derived phytochemicals, functional amino acids, organic acids, and other functional additives that pose no potential threat to livestock quality [6,7,8]. At present, research into such alternatives mainly focuses on the application of probiotics in fermented feed and how animal gut health and production performance can be maintained and improved with fermented feed. It has been reported that the fermentation of raw feed materials after inoculation with beneficial microorganisms can decompose organic macromolecules such as proteins and lipids into small molecules such as organic acids; this increases the feed’s nutritional content, allows it to be more easily absorbed by livestock and poultry, and promotes animal intestinal health, thereby improving the growth performance and health of livestock and poultry [9].

Oxidative stress [10] refers to the excessive production of highly reactive molecules, such as reactive oxygen species (ROS) and reactive nitrogen radicals (RNS), which exceeds the scavenging capacity of antioxidants, resulting in the disruption of the balance between the oxidative system and the antioxidant system, and the occurrence of damage to cells and tissues. When animals are faced with external environmental pressure, disease, feed discomfort, transportation, or other adverse stimuli, the body, especially the intestines, may experience oxidative stress, resulting in damage to cell membranes, abnormal organelle functions, the oxidation of DNA, proteins, and lipids, and even the induction of a series of adverse physiological and pathological changes such as inflammatory reactions. These reactions, in turn, affect the health and performance of the animal, in ways such as by reducing immunity, slowing growth rates, and reducing the reproductive capacity. Therefore, the effective control of oxidative stress is essential to maintaining animal health and improving breeding efficiency [11]. Research has shown that the supplementation of fermented feed can effectively help maintain the stability of the intestinal environment and reduce the adverse effects of oxidative stress [12,13]. For example, Jairath et al. [14] found that fermentation also resulted in a significant increase in antioxidant activities. Liu et al. [15] showed that fermented mixed feed was able to regulate the gut microbial community and metabolism of finishing pigs. Hu et al. [16] found that fermented tea residue significantly improved the antioxidant capacity of a juvenile largemouth bass.

Although unconventional feed raw materials have limited nutritional value and contain anti-nutritional factors, they can be transformed into unconventional fermented feed with a high nutritional value after processing via fermentation and other technologies. After fermentation, such unconventional feed not only improves the nutritional value and digestibility of conventional feed, but also reduces the oxidative stress of animals [17] and promotes improvements in livestock and poultry health and production performance [18]. It supports the production of antibiotic-free livestock and poultry products, while expanding feed resources and alleviating raw material shortages. Unconventional fermented feed maintains intestinal health by producing antioxidants and probiotics, and enhances immune function with bioactive substances [19], which is of great significance for achieving antibiotic-free animal husbandry [20], alleviating resource shortage and coping with oxidative stress. Increasing feed diversity is helpful in improving the overall growth performance of livestock and poultry, reducing environmental pollution, and promoting the green development of animal husbandry. In addition, the antioxidants in unconventional fermented feeds can improve the antioxidant capacity of animals and reduce the damage caused by free radicals, thereby further improving the health and production efficiency of animals. These antioxidants may include vitamins, enzymes, peptides, or other small molecule compounds that can neutralize free radicals in the body, reduce oxidative stress, and protect cells from damage. At present, research into the mechanism of fermented feed’s impact on oxidative stress in the intestinal tract is not sufficient, and the evaluation system for such impacts needs to be improved upon. As different fermentation strains, methods, and conditions will affect the quality of the feed, thereby affecting the growth performance and meat quality of animals, it is necessary to optimize the fermentation process and further study its impact on intestinal health, so as to provide scientific support for the application and development of unconventional fermented feed.

## 2. Types and Applications of Unconventional Feed

The sources of unconventional feed are very extensive, including, but not limited to, grain and oil processing by-products, livestock and poultry processing by-products, aquatic product processing by-products, and other industrial processing by-products. Unconventional raw materials are abundant resources, but their application in animal feeding is limited due to their high amounts of anti-nutritional factors and toxins [21,22]. Therefore, improving the quality of unconventional raw materials and elevating their utilization rate in animal feeding are important topics of current scientific feed research. Current commonly used unconventional raw materials such as wheat bran, rice bran, bean dregs, distiller’s grains, sweet potatoes, straw, and other processing by-products require physical processing, chemical treatment, or microbial fermentation to decompose crude fiber and increase their feed value. Anti-nutritional factors and toxins in unconventional fermented feed can be reduced in a number of ways. During fermentation, microorganisms and enzymes that digest anti-nutritional factors such as phytic acid and cellulose are produced [23], while high-temperature treatment helps to destroy the structure of some toxins. In addition, regulating the pH and microbial metabolism during fermentation can also reduce the toxin content in feed [24]. Physical treatments such as filtration and sedimentation can also be used to reduce toxin levels [25]. By combining these methods, unconventional fermented feed can be safely provided to animals while improving their nutrient availability and health. Table 1 shows the positive effects of the fermentation of unconventional feed on animal health, nutrition and performance, and antioxidant aspects.

### 2.1. By-Products of Grain and Oil Processing

The utilization of grain and oil processing by-products in animal feed is one of the important links in animal husbandry production. These by-products include rice, wheat, corn, beans, rapeseed, camellia seeds, and other by-products produced in the process of grain and oil processing, such as rice husks, wheat bran, corn stalks, bean dregs, etc. They may be considered unconventional in conventional feed production [35], but they are actually rich in nutrients [36] and can be used in animal feed production through a series of processing and utilization steps [37]. First of all, these by-products are properly processed, such as through milling, crushing, etc., to improve their structure and digestibility and increase their utilization. They can then be used directly as raw materials for animal feed. For example, high-fiber by-products such as wheat bran and corn stover can be used as crude fiber sources [38,39], while protein-rich by-products such as rice bran and okara [40] can be used as protein sources. In addition, some grain and oil processing by-products can also be used as feed additives. For example, cellulose-rich by-products such as rice husks and okara can be used as fiber additives to help improve the digestive health of animals [40]. Zhen et al. [41] found that the content of sugar and glycoside derivatives such as crude protein and D-threitol could be significantly increased by fermenting corn stover with Aspergillus niger, thereby enhancing the antioxidant capacity and nutritional value of fermented corn stover. Finally, these by-products can be blended with other conventional feed ingredients to form a balanced and nutritious feed formulation [42]. Xia et al. [43] showed that with the gradual increase in the proportion of peanut meal replacing soybean meal, the egg production of laying ducks increased, while the weight of the eggs and feed consumption decreased. Valenca et al. [44] found that when peanut meal replaced soybean meal as the feed, the content of oleic acid, pentadecanoic acid, heptadecanoic acid, and eicosapentaenoic acid in lamb meat increased, while the content of palmitic acid decreased, and the total amount of high-cholesterol fatty acids also decreased. These results suggest that peanut meal can improve the lipid profile of lamb meat. Wang et al. [45] found that replacing the 600 g/kg portion of soybean meal in the feed with mixed vegetable proteins (including rapeseed meal, cottonseed meal, and peanut meal) had no adverse effect on the growth performance of Yellow River carp. Azad et al. [46] showed that when soybean meal was partially replaced with 5% fermented cassava residue, piglets could increase catalase, glutathione peroxidase, and hepatic superoxide dismutase levels.

### 2.2. By-Products of Livestock and Poultry Processing

Livestock and poultry processing by-products refer to the by-products produced during the slaughtering and processing of livestock and poultry, usually including bones, internal organs, fur, blood, and feathers [47,48]. These by-products are rich in nutrients and can be used to make various types of feed, providing comprehensive nutritional support for the animals. For example, bones are rich in minerals such as calcium and phosphorus, which can be used as a supplement to minerals in feed to make bone meal or bone pulp [49], offal is rich in protein and fat, which can be used as raw materials for high-protein feed, and fur can be processed into high-protein animal fur powder as a protein source for animal feed [50]. Suryadi et al. [51] used rice-water microbial fermentation technology to transform snail meat into a new probiotic product that can be used for feed supplementation.

### 2.3. By-Products of Aquatic Products Processing

Aquatic product processing by-products are the by-products produced during the processing of fish, shrimp, shellfish, and other aquatic products [52], including fish bones [53], fish skins, shrimp shells, etc. [54]. These by-products are rich in nutrients, such as proteins, fats, minerals, etc., and have high nutritional value. These by-products can be made into powdered or granular feed ingredients through appropriate processing, such as drying, crushing, etc. Such feed ingredients can be used as an important source of protein and fat in animal feed, helping to improve the nutritional level of animals, promote their growth and development, and improve their health. Zhu et al. [55] found that the shrimp by-product astaxanthin extract had a positive effect on the antioxidant activity and color difference of readymade shrimp surimi products, which could be used as a potential multifunctional additive for the development of shrimp surimi products.

### 2.4. Other Processing By-Products

Other industrial processing by-products are by-products produced in some other industrial production processes, such as food processing by-products, wood processing by-products, etc., including mulberry leaves, moringa, chicory, arbor, seaweed, forestry by-products, etc. The crude protein content of mulberry leaves is as high as more than 16%, and mulberry leaves is also rich in a variety of bioactive substances such as flavonoids, alkaloids, polysaccharides, and polyphenols, which makes mulberry leaves have a high medicinal value [56]. Jiayu et al. [57] showed that the feed supplemented with 2% mulberry leaf powder could modify the serum metabolites of piglets, enhance the antioxidant capacity, and reduce the content of potential intestinal pathogens. Studies have shown that chicory can enhance the expression of molecular proteins, folded proteins in proteins, and antioxidant proteins in the cecum and colon of growing pigs [58], optimize the levels of odorin and androstenone in boars, and effectively reduce odor in boars [59]. Chen et al. [60] have shown that feeding piglets xylo-oligosaccharides can reduce malondialdehyde levels and catalase activity in the serum, thereby reducing oxidative damage to the body.

## 3. The Effects and Mechanism of Fermentation on the Improvement of Unconventional Feed Quality

**Figure 1:**
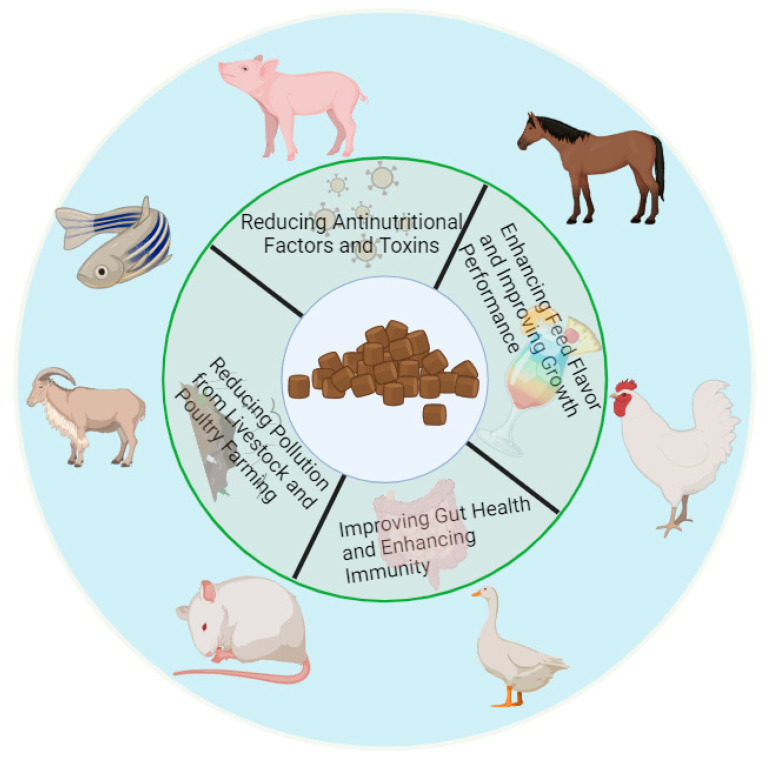
Expected characteristics of unconventional feed after fermentation. After fermentation, the anti-nutritional factors and toxins present in unconventional feed are greatly reduced, and the flavor substances of the feed are significantly increased. By eating fermented feed, animals can significantly improve their growth performance, improve their intestinal health, enhance their immunity, and reduce environmental pollution in the process of livestock and poultry breeding.

Unconventional feed comes from a wide range of abundantly produced sources. The development and utilization of unconventional raw materials can not only alleviate the problem of food security, but also serve as a way to consolidate the effect of poverty alleviation. As mentioned above, unconventional raw materials have the advantages of low environmental requirements, a wide growth range, rich nutritional value, and high total output. Nowadays, through the use of microbial fermentation technology, anti-nutritional factors and other substances in unconventional raw materials can be degraded to improve the materials’ nutritional value. Figure 1 shows the expected characteristics of unconventional feed after fermentation, which can effectively reduce the amount of anti-nutritional factors and toxins and increase the flavor substances in raw feed. The antioxidant application of unconventional fermented feed in animal production is a key measure. This type of feed protects animal cells from oxidative damage by providing a rich source of antioxidants [61] such as vitamin C, vitamin E, and polyphenolic compounds that effectively inhibit the production of free radicals. In addition, the preparation of unconventional fermented feed may degrade the anti-nutritional factors and toxins in feed, reduce the load of oxidative stress in animals [62], and further reduce the incidence of oxidative damage. These antioxidants are also able to enhance the immune function of animals, making them more resistant and thus reducing the risk of disease. It is worth noting that unconventional feed has stronger antioxidant activity after fermentation treatment [63], which can not only maintain the feed’s nutrient integrity and extend its shelf life, but can also improve its palatability, digestion, and absorption efficiency, ultimately improving the overall health and production performance of animals. Therefore, the antioxidant application of unconventional fermented feed in animal production is of great significance for improving the sustainable development of the aquaculture industry.

### 3.1. Reductions in Anti-Nutritional Factors and Toxins

Unconventional fermented feed uses a variety of effective measures to reduce anti-nutritional factors and toxins. Through microbial action [64], enzyme action [65], and acid base treatment, the microflora and enzymes in the fermentation process can decompose, transform, and reduce the contents of anti-nutritional factors and toxins. At the same time, proper heat treatment [66] and the screening of raw materials can further reduce the presence of these harmful substances. The process of microbial fermentation can effectively decompose anti-nutritional factors and toxins in feed [67], thereby reducing their content, and has the advantages of high treatment efficiency, no reagent residue, safe use, and less nutrient loss, so fermented feed has been widely used in actual livestock and poultry production. Fermentation improves not only the nutritional value, but also the safety of feed, making it more suitable for the digestion and absorption of livestock and poultry. Yeast cell walls can adsorb mycotoxins such as aflatoxin B1, reduce the absorption of mycotoxins by the digestive tract, and protect animals from the toxic effects of mycotoxins [68]. Liu et al. [69] found that microbial fermented feed could further improve the growth performance and immune indexes of offspring mice. Wang et al. [70] showed that fermented kelp residue can promote the growth of sea cucumbers, enhance the antioxidant capacity, and maintain the stability of intestinal flora.

### 3.2. Increases Feed Flavor and Improves Growth Performance

Fermentation produces a large number of organic acids, small peptides, amino acids, enzymes, and various metabolites that increase the natural flavor of feed, improve the palatability of feed, and promote the appetite of animals [71]. For example, lactic acid, butyric acid, acetic acid, propionic acid, and other organic acids [72] can reduce the pH value of feed and inhibit the growth of harmful microorganisms; small peptides and amino acids [73] provide nutrients that help animals grow and develop; and phytase, cellulose, and other enzymes [74] can help to decompose complex polysaccharides and proteins and improve the digestion and absorption rate of feed. Taken together, these effects promote the digestion and absorption of nutrients and improve animal growth performance [75,76]. Yeast produces alcohol and other volatile compounds with a distinctive wine aroma during fermentation. Lactic acid bacteria produce a large amount of lactic acid, which has a sour aroma, during fermentation. Both different types of fermented feed can increase the appetite of animals while also having an increased nutritional value. Studies have shown that fermented feed can increase the contents of unsaturated fatty acids and amino acids in the muscles of fattening pigs during the growth period, enhance the antioxidant capacity, and improve pork quality [77,78,79]. Liu et al. [80] found that fermented mixed feed could up-regulate the expression of unsaturated fatty acid synthesis (acetyl-coenzyme A acyltransferase 1 and fatty acid desaturase 2), enhance the antioxidant capacity, and improve meat quality in fattening pigs. Zhu et al. [28] found that fermented feed could improve the growth performance, immune organ development and ability, and intestinal antioxidant capacity of broiler chickens.

### 3.3. Improves Intestinal Health and Strengthens Immunity

Unconventional fermented feed plays an important role in improving gut health and boosting immunity. First of all, the beneficial microorganisms and metabolites produced by the fermentation process help to maintain the intestinal microecological balance, inhibit the growth of harmful bacteria, and reduce the invasion of pathogenic bacteria to the intestine, thereby improving intestinal health [81]. Secondly, the bioactive substances in fermented feed, such as organic acids, enzymes, and peptides, can promote the growth and repair of intestinal mucosal cells, enhance intestinal barrier function, and prevent the penetration of harmful substances [82]. In addition, the bioactive ingredients rich in fermented feed can also regulate the function of the immune system, promote the generation and activity of immune cells, and improve the resistance and immunity of animals [83]. Beneficial microorganisms in fermented feed can improve the structure of the intestinal microbial communities of animals [84,85], promote intestinal health, and improve the digestion and absorption of nutrients. Microbial fermented feed can stimulate the development of immune organs, improve immune function, and increase the weight of immune organs, thereby strengthening the immunity of animals [86,87,88]. Studies have shown that fermented feed has a positive impact on improving the production efficiency of laying hens, improving egg quality and gut health [89,90]. Lactation is an important stage of pig reproduction [91], and Wang et al. [92] found that the addition of 15% *Bacillus subtilis* and *Enterococcus faecalis* to a fermented corn–soybean meal mixture significantly increased the average daily gain (ADG), litter gain, and individual piglet gain during lactation. With the addition of *Bacillus mesenterica*, *Enterococcus faecalis*, and *Clostridium butyricum* to fermented feed, the productivity of sows and offspring was effectively improved. In addition, Grela et al. [93] showed that the fermented rapeseed had a positive effect on nutrient digestibility during lactation in sows. Qiang et al. [94] found that the fermentation of wolfberry through modern fermentation technology can increase the levels of a variety of nutrients and bioactive components, as well as improve the antioxidant capacity.

### 3.4. Reduces Pollution from Livestock and Poultry Farming

Unconventional fermented feed plays an important role in reducing pollution from livestock and poultry farming. First of all, the use of unconventional raw materials as the main component of fermented feed can effectively use resources such as agricultural and sideline products and aquatic product by-products, reduce the demand for traditional raw feed materials, and thus reduce resource consumption and environmental pressure in the agricultural production process [27]. Secondly, the microorganisms and enzymes in the fermentation process can degrade organic matter in organic waste, such as straw and manure, which reduces the emission of organic waste and reduces the pollution of livestock and poultry breeding to the environment [95]. In addition, the antioxidants and bioactive components in fermented feed can also improve the digestion and absorption capacity of animals, reduce the emission of nutrients in manure, and further reduce the pollution of water and soil by livestock and poultry farming. Pathogens in feces can pollute the farm environment, including the water and soil, which is harmful to human and animal health [96]. The main pathogen on farms is Enterobacteriaceae, which leads to impaired barrier function and malnutrition by colonizing the intestinal mucosa of pigs [97]. The use of fermented feed in livestock and poultry farming can significantly reduce environmental impacts. This is mainly due to the fact that fermented feed can reduce the discharge of manure, eliminate the foul smell of manure, and reduce the number of mosquitoes and flies, thus effectively reducing environmental pollution. A study by Hăbeanu et al. [98] showed that when a mixture of puffed flaxseed and walnut powder (50:50) was added to a pig’s diet, the amount of nitrogen excreted by the pig was significantly reduced. Additionally, feeding poultry with probiotic fermented feed was shown to reduce nitrogen and phosphorus emissions in manure.

## 4. Unconventional Fermented Feed Improves Oxidative Stress by Regulating Gut Health

**Figure 2:**
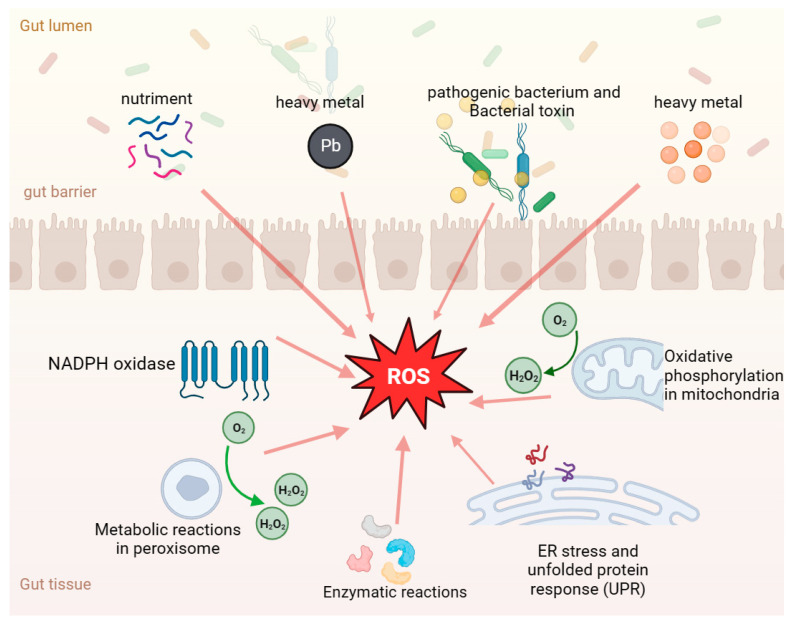
Development of ROS in the gastrointestinal tract. The orange arrows indicate that ROS can be caused by a variety of factors, such as intestinal nutrient stimulation, antigens, pathogenic bacteria, mycotoxin contamination, and heavy metal pollution, as well as autometabolism by-products such as peroxidase and oxidative phosphorylation.

The highly intensive development of modern animal husbandry is prone to result in oxidative stress in animals [99], which can cause damage to intestinal health, including destroying intestinal mucosal structures and affecting the absorption function of the intestine, thus affecting the growth performance of animals. The development of ROS in the gastrointestinal tract is shown in Figure 2. Preventing oxidative stress in animals is a significant issue. The use of probiotic fermented feed to feed animals has been recognized and widely used in animal husbandry production [100]. Many studies have shown that fermented feed can affect the stability of the gut microbiota and the proper function of the gut [101,102]. Moreover, dietary fiber in fermented feed plays an important role in regulating the lipid metabolism and antioxidant stress response, and it has significant effects in improving cardiovascular and cerebrovascular diseases, adjusting the structure of intestinal flora, and affecting the energy balance through the gut microbiota gut–brain axis, thereby having a positive impact on health [103,104,105]. Fermented feed plays a key role in antioxidant activity [22,106,107] as it is rich in antioxidants (e.g., vitamin C, vitamin E, and polyphenols), and some microbial metabolites produced during fermentation also have an antioxidant capacity, which helps maintain the redox balance in the body [108]. In addition, other significant advantages of fermented feed are that it improves the utilization rate of feed nutrients, effectively improves the nutritional level of animals, and enhances the ability to resist oxidative stress [109,110]. Indeed, the use of fermented feed not only provides antioxidants and microbial metabolites, but also promotes the absorption of nutrients, which is an important breeding strategy that is beneficial to animal health and physiological balance.

### 4.1. Gut Health and Oxidative Stress

**Figure 3:**
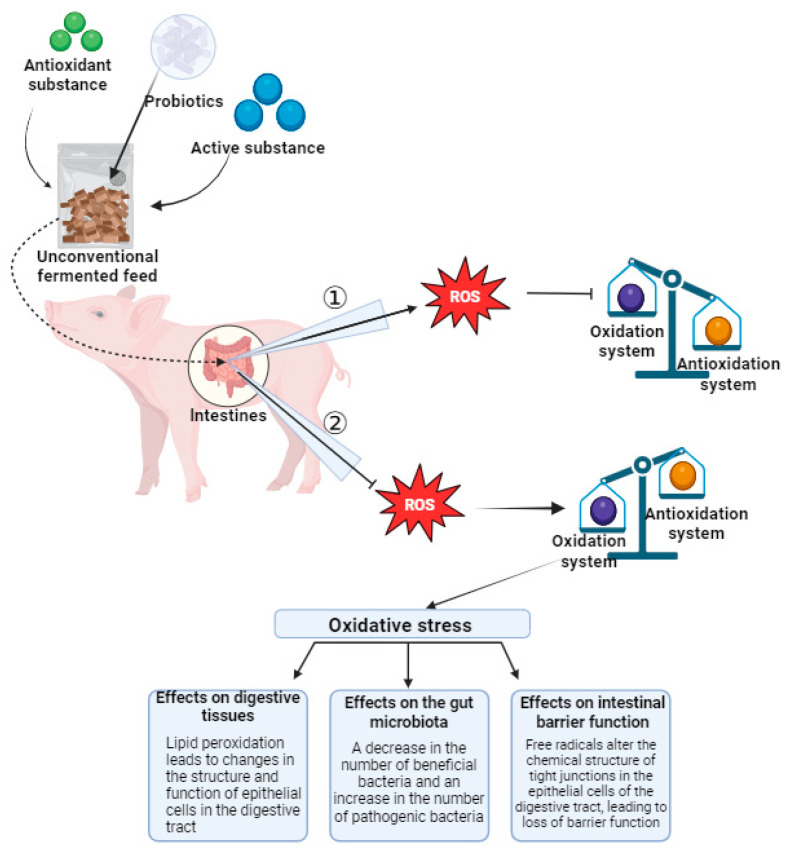
Unsaturated fatty acids in membranes of the digestive tract epithelium are prone to react with free radicals. Beneficial gut bacteria have a reduced ability to produce metabolites such as antioxidant enzymes or SCFA. The changes of free radicals in the chemical structure of tight junctions in epithelial cells of the digestive tract lead to the loss of barrier function. Arrow ① indicates that feeding animals with unconventional fermented feed can affect the development of ROS, inhibiting the impact of ROS on the oxidative–antioxidative balance system. Arrow ② indicates that animals not fed with unconventional fermented feed are unable to inhibit ROS, thus disrupting the oxidative–antioxidative balance. When the oxidative–antioxidative balance is disrupted, animals experience oxidative stress, which adversely affects digestive tissues, intestinal microbiota, and intestinal barrier function.

Gut health is an important foundation for animal health and growth performance, and the gut is one of the most important sources of oxygen free radicals, which can be produced in the intestinal lumen and intestinal mucosa [111]. When the intestinal environment changes, intestinal oxidative stress may be triggered, which may result in changes in the nutrient content, increases in the number of antigens, the invasion of pathogenic bacilli, mycotoxin contamination, and heavy metal contamination. These factors can upset the balance of microbes in the gut, leading to an increase in harmful microflora, which can trigger intestinal inflammation and damage. In addition, the metabolites and free radicals produced during autometabolism may also cause damage to intestinal cells, leading to the occurrence of oxidative stress. Oxidative stress causes an imbalance in the redox balance within cells, increases the production of free radicals, and leads to the lipid peroxidation of cell membranes, the oxidation of proteins, and damage to DNA [112,113]. The damage of ROS to the intestine is affected by a variety of factors, such as the amount of free radicals and the antioxidant capacity of the body. In general, the ROS produced by the body are quickly removed by the body’s antioxidant system, which helps to maintain oxidative homeostasis and avoid oxidative damage to the body. However, if too much ROS are produced or the antioxidant system is less able to scavenge ROS, the homeostasis between oxidation and antioxidation will be disrupted. In this case, ROS cannot be removed in time and will accumulate in large quantities in the body, which may eventually lead to oxidative stress damage, especially in the intestines [114]. Studies have shown that macromolecular degradation by-products such as respiratory metabolites, lipids, and proteins, metabolites produced by macrophages when they engulf viruses, and the cytochrome P450 system are the main sources of ROS production in the gut [115,116,117]. When an oxidative response is triggered, the regenerating digestive tract epithelial cells are particularly sensitive to free radicals and the unsaturated fatty acids in the cell membrane easily react with these free radicals, resulting in lipid peroxidation, a decline of microbial diversity in the intestinal flora, a decrease in the number of beneficial bacteria, an increase in the number of pathogenic bacteria, a reduction in the ability of beneficial bacteria to produce metabolites such as antioxidant enzymes or short-chain fatty acids (SCFAs), and the change of the free radicals in the chemical structure of the tight junction of digestive tract epithelial cells, which results in the loss of the barrier function; all of these processes are significant hidden dangers to animals. At this point, an animal can reduce the damage caused by oxidative stress by ingesting substances with antioxidant activity (Figure 3). Wang et al. [118] found that spermidine could improve the intestinal health of a Sichuan white goose by improving its intestinal morphology, increasing the antioxidant capacity, and regulating intestinal microbiota structures. Liu et al. [119] showed that cysteine could inhibit the intestinal nuclear factor-κB (NF-κB)/inhibitor of κB kinase/inhibitor of nf-κB signaling pathway and pro-inflammatory cytokine mRNA levels, as well as increase the diversity and relative abundance of intestinal microbiota in golden pomfret. Li et al. [120] found that probiotics inhibited inflammation and oxidative stress by improving intestinal SCFA and inhibiting the p38 mitogen-activated protein kinase/NF-κB pathway in colon tissue.

### 4.2. Effects of Unconventional Fermented Feed on the Morphological Structure of Intestinal Mucosa

**Figure 4:**
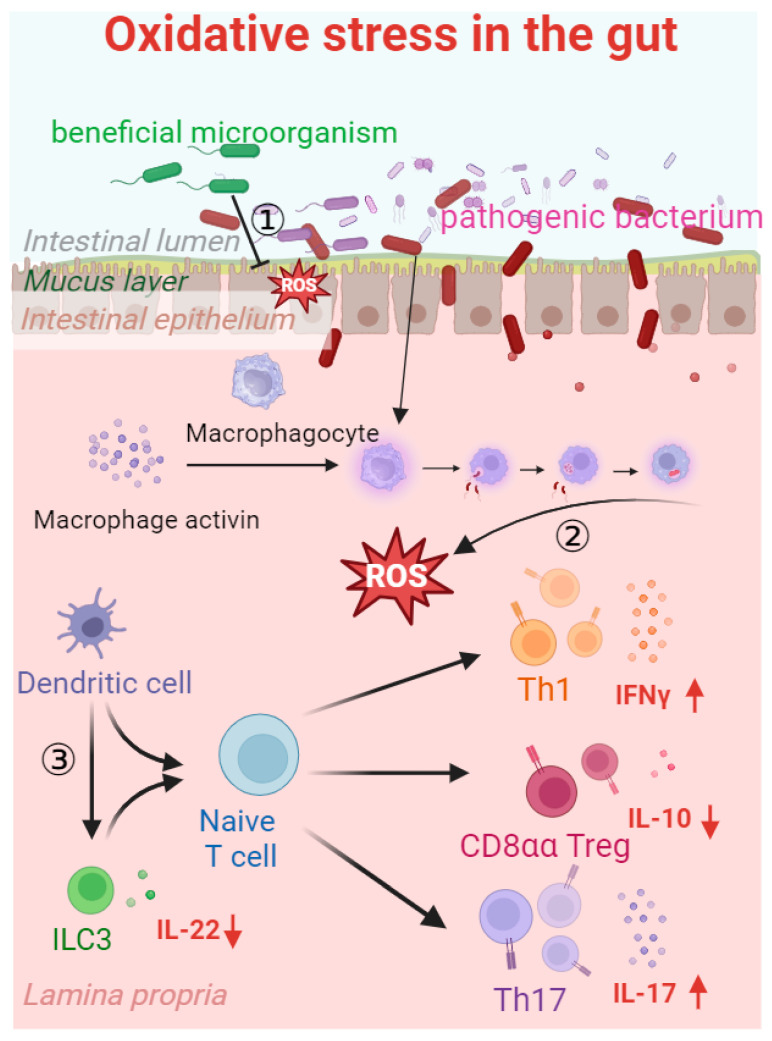
Oxidative stress in the gut. Pathogenic bacteria enter the internal environment by damaging the gaps between intestinal epithelial cells, triggering an inflammatory response. Inflammatory factors activate macrophages to produce ROS during phagocytosis, which strengthens the inhibition of beneficial bacterial colonization and biological functions. In the case of impaired barrier function, group 3 innate lymphoid cells (ILC3 cells), CD8aa cells, Th17 cells, and Th1 cells are stimulated, thereby reducing the secretion of interleukin-22 (IL-22) (↓) and interleukin-10 (IL-10) (↓) and increasing the secretion of interferon-γ (IFNγ) (↑) and interleukin-17 (IL-17) (↑). Inhibition line ① indicates the definite vegetation inhibition of beneficial microorganisms in the intestine. Arrow ② indicates that ROS are produced when harmful bacteria invade and macrophages initiate phagocytosis. Arrow ③ indicates that dendritic cells are influenced by oxidative stress, leading to the stimulation of ILC3 cells, CD8aa cells, Th17 cells, and Th1 cells, resulting in the decreased secretion of IL-22 and IL-10 and the increased secretion of IFNγ and IL-17.

Changes in intestinal morphology (villi height and crypt depth) are the main causes of functional alterations and decreased intestinal absorptivity. When stress is generated, the level of ROS in the gut is elevated and the microbiome balance is disrupted (Figure 4), which also causes the increased mitosis of intestinal cells and the increased depth of the intestinal crypts. When the rate of intestinal cell migration to villi increases, so does the rate of apical intestinal cell shedding, which leads to an increased loss of mature intestinal cells, thereby reducing enzyme activity and absorption [111]. This may increase the sensitivity of the small intestine to pathogenic microorganisms, such as *E. coli*, further exacerbating damage to intestinal tissue. Fermented feed can effectively neutralize free radicals in the intestine through the antioxidants and microbial products it contains, slow down the production of oxidative stress, improve the length and width of villi, inhibit inflammation, reduce intestinal permeability [121], and help maintain the stability of the body’s intestinal environment and intestinal morphology [122].

The effects of unconventional fermented feed on the intestinal health of animals can be seen in a number of ways. First, by influencing the gut microbiota, unconventional fermented feed is able to regulate the structure and composition of the gut microbiota of animals at different stages of growth and to improve the digestibility and overall gastrointestinal function of animals, thereby reducing the risk of diarrhea, helping to maintain a healthy state, and improving the growth performance and antioxidant capacity [26]. Secondly, unconventional feed grows rich in beneficial microorganisms and nutrients after fermentation, which can improve the morphological structure of the intestinal mucosa, increase the height and area of intestinal villi, and further maintain the self-stability of the intestine [123]. The ratio of villi height to crypt depth (V/C) is directly proportional to the body’s ability to absorb nutrients [124]. Dietary supplementation with Bacillus subtilis has been shown to promote the development of small intestinal villi [125]. Sugiharto et al. [126] found that supplementing a basic diet with 10~15% fermented banana peel and the same amount of corn could significantly increase the ileal villi height of Roman broilers. Liu et al. [127] found that feeding layers with fermented mixed feed significantly reduced the depth of crypts in the duodenum and jejunum and increased the ratio of villus height to crypt depth. Wu et al. [128] showed that the duodenal villi height/crypt depth ratio increased after broilers were fed fermented soybean meal (FSBM). Yu et al. [129] found that a level of 6.73~20.18% fermented cottonseed meal in a meat goose diet significantly increased the villi height of the jejunum. Feeding *Bacillus subtilis* was shown to increase the length of intestinal villi in *Nile tilapia* [130]. Feeding Nile tilapia with a bacillus-rich diet resulted in an increase in the number of goblet cells [131].

### 4.3. Effects of Unconventional Fermented Feed on Intestinal Barrier and Absorption Function

The bioactive ingredients and beneficial microorganisms in fermented feed help to enhance the integrity and tightness of the intestinal mucosa, prevent the penetration of harmful substances, reduce the invasion of pathogenic microorganisms, and protect the intestines from damage [81]. Enzymes and metabolites in fermented feed can promote the decomposition and release of nutrients, improve nutrient availability, and thus enhance the ability of animals to digest and absorb nutrients. In addition, fermented feed is rich in antioxidants, which can scavenge free radicals, reduce the impact of oxidative stress on the intestinal barrier and absorption function, protect the health of intestinal mucosal cells and tissues, and maintain normal cell and tissue function [132]. The intestinal mucosa, as the largest interface between the body and the external environment, is easily disturbed by factors such as pathogenic microorganisms, pro-inflammatory factors, and antigens. The mechanical barrier of the intestinal mucosa can prevent damage to the small intestine caused by foreign substances and reduce the permeability of the intestinal mucosa, thereby maintaining the health of the intestine. ROS ultimately damage the intestinal biological barrier by affecting the adhesion and colonization of microorganisms and intestinal epithelial cells and the digestion and metabolism of nutrients between microorganisms and hosts in the gut. The various digestive enzymes produced by probiotics in fermented feed during the fermentation process can decompose macromolecular substances in raw feed materials, such as starch, protein, and cellulose, into small molecule substances for better digestion by animals [133]. These digestive enzymes can also be used as a supplement to the digestive enzymes of animals’ digestive tracts to further break down nutrients, thereby improving the digestion and absorption of nutrients [134,135,136] and improving the growth performance [137]. When piglets are weaned separately from sows, physiological, environmental, and social stresses can lead to multiple stress responses. These stressors include oxidative stress, barrier dysfunction, and disturbances in the gut microbiota. In addition, weaned piglets are susceptible to immune stimulation due to imperfect digestive systems, low digestive enzyme activity, low immunity, etc., resulting in immune imbalance, oxidative stress, diarrhea, and even death. These stress responses may disrupt the development of the intestinal immune system of piglets, inhibiting their growth and development [138]. Xin et al. [139] showed that liquid fermented feed could significantly increase the daily weight gain of weaned piglets, as well as the abundance of lactic acid bacteria in cecal chyme and the level of acetic acid in colonic chyme. Wang et al. [140] reported that fermented feed improved the hematologic profile and serum concentrations of the total protein, albumin, and globulin in weaned piglets. Wang et al. [141] showed that FSBM could alleviate diarrhea symptoms in weaned piglets by modulating the cecal microbiota composition. Guo et al. [142] found that when 6% compound probiotic fermented feed was added to the basal diet of laying hens, the relative mRNA expression level of Occludin in the jejunal mucosa could be significantly increased, and the physical barrier function of the gut of laying hens could be improved. Wang et al. [143] treated piglets with the Lactobacillus plantarum strain ZLP 001 and found that the density of *Clostridium* spp_1 cells associated with epithelial inflammation in the experimental group was reduced, and the expression and secretion of pro-inflammatory cytokines IL-6 and TNF-α were down-regulated, which strengthened the intestinal barrier function and antioxidant capacity of piglets.

### 4.4. Effects of Unconventional Fermented Feed on Gut Microbiota

The effect of unconventional fermented feed on intestinal microbiota is significant. Its functions are mainly reflected in regulating the structure and composition of microflora, increasing the number of beneficial microorganisms, promoting the diversity of microflora, and improving the metabolic function of microflora [132]. Through these effects, fermented feed can maintain the balance and stability of the intestinal flora, promote the growth and reproduction of beneficial bacteria, and inhibit the reproduction of harmful bacteria, thereby improving the intestinal health of animals. This combined effect helps to improve the digestion and absorption capacity of animals, enhance immunity, and reduce the occurrence of intestinal diseases such as diarrhea [144]. Fermented feed is rich in organic acids, amino acids, and small peptides after decomposition; these components have a role in regulating dysbiosis [145,146]. Under normal circumstances, the intestinal tract is in a state of microecological balance, and beneficial bacteria such as lactic acid bacteria and bifidobacteria in the intestine adhere to the intestinal epithelial cells to produce bacteriocins, organic acids, and other bacteriostatic substances to inhibit the adhesion, colonization and invasion of pathogenic bacteria. Intestinal microbiota are in a state of reciprocal symbiosis with their host. For example, the transplantation of fecal bacteria has been used to clinically treat diseases caused by intestinal homeostasis imbalance [147,148]. Probiotic supplementation can help alleviate intestinal diseases (such as intestinal inflammation and intestinal cancer diseases), enhance intestinal function, and improve antioxidant capacity [149]. Studies have shown that mice can be supplemented with *Lactobacillus gasseri* to produce SCFAs [150,151] and inhibit the development of intestinal inflammation-related cancers [152]. These microorganisms can effectively improve the structure of intestinal microbial communities, increase the number of beneficial bacteria in the intestine, reduce the number of harmful bacteria, and improve intestinal health through fermented feed [153]. Xin et al. [139] fed fattening pigs with fermentation broth, and the results showed that the final body weight and ADG of the fattening pigs significantly increased, the total volatile fatty acid content showed an upward trend, and the total intestinal digestibility increased. O’Meara et al. [154] found that the liquid feeding of fermented cereals to fattening pigs could increase the abundance of lactic acid bacteria, reduce the abundance of Enterobacteriaceae bacteria, and improve the composition of intestinal microbiota. Guo et al. [142] showed that adding 6% compound probiotic fermented feed to a diet could regulate the microbial structure of the cecum and improve the intestinal health of laying hens.

### 4.5. Effects of Unconventional Fermented Feed on Immune Function

**Figure 5:**
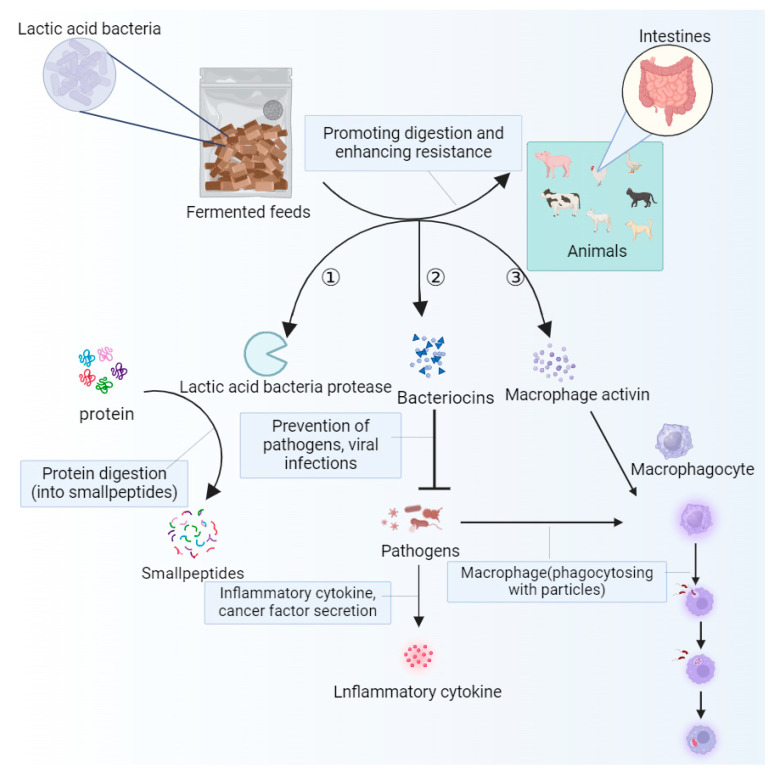
Lactic acid bacteria promote digestion, inhibit microorganisms, and activate the body’s immune response mechanism. Arrow ① indicates that lactic acid bacteria secrete lactobacillus proteases to break down proteins into peptides; arrow ② indicates that lactic acid bacteria secrete bacteriocin, inhibiting the growth of pathogenic microorganisms, reducing the expression of inflammatory factors, and activating the phagocytic function of macrophages; arrow ③ indicates that lactic acid bacteria secrete macrophage activators, activating the phagocytic function of macrophages and generating immune responses.

Immunomodulatory effects can promote the production and activation of immune cells, enhance the body’s immune response, and reduce the risk of animal disease [132]. Secondly, the anti-inflammatory components in fermented feed can inhibit the occurrence of inflammatory reactions, reduce the burden on the immune system, and protect the body’s tissues from damage. In addition, fermented feed can also promote the development and function of immune organs, as well as enhance the overall function of the immune system. Most importantly, it is rich in antioxidants, which can scavenge free radicals, reduce oxidative stress damage to the immune system, and improve the stability and activity of the immune system [106]. The use of unconventional fermented feed not only increases the contents of immunoactive substances such as cytokines, antibodies, and lysozymes, but also improves the immune capacity of animals by stimulating the development of immune organs, improving immune function, and increasing the weight of immune organs [155], as well as ameliorating the damage of oxidative stress to the intestine [86,87,88]. There are many different microbes in the gut that are involved in different immune defense mechanisms and influence the body’s immune response. Under intestinal oxidative stress, the contents of immunoglobulin A, immunoglobulin G, and immunoglobulin M in the intestine decrease, the number of T cells decreases, and cellular immunity is abnormal. The use of unconventional fermented feed can effectively alleviate the occurrence of this situation. The SCFAs produced during the fermentation of unconventional feed are involved in the regulation of the intestinal mechanical barrier of livestock and poultry, improving the activity of immune substances [156] and reducing the content of harmful bacteria. Studies have shown that the number and activity of immune cells are significantly enhanced when animals ingest microbial fermented feed [157,158]. According to Taranu et al. [159], feeding fermented cassava residue can increase the serum immunoglobulin content in pigs, improving humoral immune activity, and thus enhancing disease resistance. Wu et al. [128] found that the addition of FSBM to broiler diets significantly increased the weight of the bursa and thymus and reduced serum alanine aminotransferase levels. The addition of fermented full-price feed significantly increased the serum levels of interleukin-1β, IL-6, and TNF-a in laying hens [160]. Feeding fermented full-price feed significantly increased the content of insulin-like growth factor-1 (IGF-1) in the sera of finishing pigs [161], promoted the expression of the IGF-1 protein in the liver, and improved growth performance [162]. Cheng et al. [163] showed that fermented feed inhibited the growth of harmful microflora such as *E. coli* and *enterococci*, while increasing the number of beneficial microflora such as *Bifidobacteria*, *Lactobacillus*, and *Ackermansi*. As shown in Figure 5, lactic acid bacteria decompose the proteins in feed through the lactic acid bacteria proteases secreted by themselves, so the proteins are converted into small molecule peptides and amino acids and the digestion and absorption of feed by livestock and poultry are improved. The bacteriocins secreted by lactic acid bacteria have the miraculous effect of inhibiting the growth of pathogenic microorganisms present in the intestines after entering the intestines of livestock and poultry. With the colonization of lactic acid bacteria in the intestine, macrophages can be activated to a certain extent, promoting the immune response of immune cells in the intestine, and improving the resistance of livestock and poultry to diseases [4]. Azad et al. [46] showed that when soybean meal was partially replaced with 5% fermented cassava residue, piglets could increase their CAT, GSH-Px, and HSOD levels, as well as enhance their antioxidant capacity.

### 4.6. Effects of Unconventional Fermented Feed on Antioxidant Capacity

Unconventional fermented feed has significant effects on the antioxidant capacity of animals as it is rich in antioxidant substances, such as vitamins, polyphenolic compounds, and organic acids, that can effectively remove free radicals in the body, regulate redox balance, enhance antioxidant enzyme activity, and improve the antioxidant capacity of animals [164]. These antioxidants not only protect cells and tissues from oxidative damage, but also improve animal immunity, reduce the risk of disease, and promote the healthy growth and performance of animals [165]. The beneficial microbial metabolites and active substances in unconventional fermented feed have the effects of promoting the activity of antioxidant enzymes, directly or indirectly affecting the activity of antioxidant enzymes, and slowing down the occurrence of oxidative stress [166,167]. Superoxide dismutase (SOD) scavenges ROS in the cytoplasm by catalyzing the disproportionation of two superoxide radicals to H_2_O_2_ and oxygen. GSH-Px and CAT [159] convert ROS to water and oxygen. MDA is one of the end products of lipid peroxidative damage by free radicals, and it can be used as a direct indicator of tissue damage caused by oxygen free radicals [168]; the higher the content, the higher the degree of cell damage and the weaker the antioxidant capacity [169]. The level of the total antioxidant capacity can directly reflect the level of antioxidant capacity in animals [170]. Lactic acid bacteria are partially lysed in the intestinal tract and release intracellular substances such as SOD and GSH-Px to reduce the cell damage caused by oxygen free radicals. Dong et al. [171] found that *Lactobacillus plantarum KLDS 1.0386* has the ability to scavenge hydroxyl radicals and lipid antioxidants through in vitro tests. Hao et al. [172] found that the use of fermented mixtures was able to increase the activities of SOD and GSH-Px in the sera and longissimus dorsi muscles of fattening pigs. Chen et al. [173] showed that microbial fermentation can alter the structural characteristics of wheat bran polysaccharides and increase their antioxidant activity. FSBM can increase SOD and CAT activities and vitamin C levels in piglet tissues [174]. As the main raw material for the industrial production of natural astaxanthin, astaxanthin can significantly reduce the levels of ROS and MDA in the sera of pigs [175]. Studies have shown that yeast extracts are able to increase the antioxidant capacity of aquatic animals [176]. Long et al. [177] showed that dietary supplementation with Saccharomyces cerevisiae increased serum SOD activity and reduced MDA levels in weaned piglets.

In conclusion, unconventional fermented feed can regulate the structure of the gut and the composition of intestinal microbiota, which can effectively improve the digestibility and overall gastrointestinal function of animals, reduce the risk of oxidative stress, and help maintain a healthy state and improve growth performance [146,178,179].

## 5. Prospects

The use of unconventional fermented feed has a positive impact on sustainable and environmentally friendly livestock practices in many ways. First of all, this kind of feed uses diversified raw materials such as agricultural and sideline products, aquatic by-products, and industrial by-products, which effectively use resources and reduce resource waste. Secondly, compared with traditional feed production, the production of unconventional fermented feed produces less environmental pollution [59] and greenhouse gas emissions, which helps to reduce the environmental load [180]. In addition, unconventional fermented feed may contain richer nutrients and bioactives than traditional feed, which can improve the growth, development rate [181], immunity, and disease resistance of animals, thereby improving breeding efficiency. Most importantly, the use of unconventional fermented feed promotes the practice of the circular economy, and the goals of waste reduction, resource conservation, and ecological environmental protection can be achieved through waste resource utilization and resource reuse, which is conducive to building a more sustainable and environmentally friendly livestock system.

The rapid development of unconventional fermented feed is important for the livestock industry, but the challenges and limitations require targeted solutions and further research.

(1) Diversified sources of raw materials: Exploring a greater variety of unconventional raw materials, such as agricultural by-products, aquatic by-products, and industrial by-products, can reduce supply instability through inter-regional and inter-seasonal diversification. The introduction of diversified sources of raw materials can not only reduce excessive dependence on a specific raw material, but can also help to improve the diversity and nutritional balance of feed.
(2) Optimization of the fermentation process: By studying and optimizing the fermentation process, such as by controlling parameters such as temperature, humidity, and PH, the stability and efficiency of the fermentation process can be improved. The introduction of automation technology and intelligent monitoring systems can be used to help reduce human error, improve production efficiency, and improve product quality.
(3) Anti-nutritional factor processing technology: The development of efficient treatment technologies, such as enzymatic hydrolysis, heat treatment, and acid–base treatment, can effectively reduce the content of antinutritional factors in raw materials. Further research into the mechanisms of different antinutritional factors in animal digestion and absorption is needed to optimize treatment techniques and ensure the nutritional value of feed.
(4) Economic benefit evaluation: A comprehensive economic benefit evaluation study should be conducted to consider production costs, animal growth performance, feed utilization efficiency, and other factors to provide a more concrete and reliable economic decision-making basis for farmers and breeders. Tailored economic assessments should be conducted for different farming scales and regional characteristics to ensure the practical feasibility and sustainability of solutions.
(5) An in-depth study of animal health and oxidative stress mechanisms: researchers should investigate the mechanism of fermented feed on the intestinal microecological balance, and explore its specific effects on animal immunity and disease resistance in order to further understand the regulatory effects of active ingredients in fermented feed on oxidative stress in animals, as well as the mechanism of their effects on the overall growth performance and meat quality.

Through in-depth research and the continuous improvement of these aspects, the challenges faced by unconventional fermented feed can be better met and its wide application and development in animal husbandry can be promoted.

## 6. Conclusions

Oxidative stress means that the oxidative substances in animals exceed the scavenging capacity of their antioxidant systems, which may have negative effects on animal health, including affecting the intestinal barrier function and inducing an inflammatory response. As a potential feed alternative to improve animal health, unconventional fermented feed may help reduce the levels of oxidative stress in animals by being rich in antioxidants, maintaining the intestinal microbiota balance, and providing nutrient support. However, they need to be carefully selected and managed to ensure that their effects on animals are positive, and they should be monitored and adjusted on a case-by-case basis.

Therefore, the exploration of the use of unconventional fermented feed to maintain the intestinal health of animals is promising. In future research, it is crucial to further improve the quality of fermented feed and optimize the technological process of fermented feed. At the same time, by studying the mechanism of fermented feed in regulating gut health and the antioxidative capacity, we can provide strong support for the development of more scientific feeding strategies, thereby promoting the overall health and performance of animals. This systematic research will help to continuously improve the application of unconventional fermented feed and promote the development of animal husbandry in a more sustainable and efficient direction.

## Figures and Tables

**Table 1:** Positive effects of the fermentation of unconventional feed on animal health, nutrition and performance, and antioxidant aspects.

Unconventional Fermented Feed Feeding					
Animal	Raw Materials	Probiotics	Regulated Items	Antioxidant Substance	References
Boer goats	*Pennisetum giganteum*	*Bacillus coagulans* preparation	Abundance of Lactobacillus and unidentified Clostridiales ↑ Anaerovibrio and Methanobrevibacter ↓	CAT, GSH-Px activities and glutathione ↑	Qiu et al. [26]
Laying hens	Corn–soybean meal wheat bran	*Bacillus subtilis* and *Saccharomyces cerevisiae*	In relative Lactobacillus, Megasphaera, and Peptococcus abundance ↑ Campylobacter abundance ↓	Immunoglobulin A, immunoglobulin M, and immunoglobulin G ↑	Guo et al. [27]
Broilers	Corn, soybean meal, corn–gluten meal, and corn dried distillers’ grains	*Lactobacillus plantarum*, *Bacillus subtilis*, and *Saccharomyces cerevisiae*	Abundance of Ruminococcaceae, Lactobacillaceae, and unclassified Clostridiales ↑ Abundance of Rikenellaceae, Lachnospiraceae, and Bacteroidaceae ↓	Acetic acid, propionic acid, butyric acid, and lactic acid ↑	Zhu et al. [28]
Laying hens	Astragalus	*Lactobacillus plantarum*	CAT, GSH-Px, superoxide dismutase and total antioxidant capacity in serum ↑	CAT ↑	Hong et al. [29]
Cobb male broilers	Corn–soybean meal	*Lactobacillus acidophilus*	Body weight, ADG, average daily feed intake, and jejunum and ileum V:C ratio at 14 d and 21 d ↑	The mRNA expression of inducible nitric oxide synthase, interleukin-8, and interleukin-1β in the jejunum ↓	Wu et al. [30]
Nursery pig	Corn–soybean meal	*Lactobacillus plantarum* and *Pediococcus acidilactici*	ADG and significantly increased fecal acetate, butyrate, and total short-chain fatty acid concentrations ↑	Short-chain fatty acid ↑	Yang et al. [31]
Berkshire pigs	Rubus coreanus	*Lactobacillus plantarum*	The mRNA expression of transcription factors and cytokines in Th1 and Treg cells ↑ The mRNA expression of T helper cell 2 and Th17 transcription factors and cytokines ↓	The mRNA expression of transcription factors and cytokines in Th1 and Treg cells ↑	Yu et al. [32]
*Cyprinus carpio*	Wheat, soybean meal, corn–gluten meal, chicken meal	*C. somerae XMX-1*, *S. cerevisiae GCC*−1, *L. rhamnosus GCC-3*, and *B. subtilis HGcc-1*	Health and production ↑	Liver anti-inflammatory factors transforming growth Factor-β↑	Zhang et al. [33]
*Juvenile olive flounder*	Garlic husks, Tuna	*Bacillus licheniformis* and *Bacillus subtilis*	Weight gain, specific growth rate, and feed efficiency ↑	Sucrose reductase↑	Fatma et al. [34]

↑ indicates an increase in substance content or enzyme activity; ↓ indicates a decrease in substance content or enzyme activity. CAT, catalase; GSH-Px, glutathione peroxidase; ADG, average daily gain; Th1, T helper cell 1; Th17, T helper cell 17.
